# Brueghel: his art and his syndrome

**DOI:** 10.1080/17571472.2017.1317405

**Published:** 2017-04-23

**Authors:** Francesco Carelli

**Affiliations:** Family Medicine, Milan and Rome, Italy

**Keywords:** Art, Brueghel’s syndrome, blepharospasm, oromandibular dystoni

The exhibition ‘Brueghel: Flemish art masterpieces’ in the Venaria Royal Palace, near Turin (September 2016–March 2017) covers the story of more than 150 years, showing the masterpieces of an exceptionally talented dynasty, living and working between sixteenth and seventeenth centuries.

## The exhibition

The exhibition is a fascinating journey into the Golden Age of Flemish Paintings of the seventeenth century, in a search for the visionary genius of as many as five generations of artists. They are able to embody as one, like as nobody before or after them, the style and trends of more than a century of history of art. While the Italian Renaissance focuses attention on the human figure’s noble and virtuous ideal through the experiences of Michelangelo, Leonardo and Tiziano, in Flanders the perspective radically changes.

Pieter Brueghel the Elder, the family’s ancestor who knew well Italian painting, addresses himself to the daily reality of human life, and investigated all its aspects without exclusion.

He dwelled on peasants’ and merchants’ shadows and vices, on living labour, but also on people’s cheeriness, often coarse and rough. It was inevitably both crude and realistic. And if in Italian Renaissance, nature is restricted to be the background for the magnificent plastic and aesthetic superiority of man; in Flemish painting, and in Brueghel’s style, nature fully takes on the role of the true protagonist of human history. It is presented with a visual wealth, an attention to detail and a compositional beauty never seen before in the history of painting.

Pieter Brueghel the Elder, and his rich progeny after him, were the inventors of a pictorial code soon to become the brand of his articulate family that since the middle of sixteenth century were active for two centuries. Brueghel’s gaze settled on simple humanity, free but at the same time the slave of its needs, continuously moving between spiritual trends and the virtuous and vicious carnal seductions.

The Brueghel are tellers of facts and stories. In their works there is a telling of true life, as peasants bent by their living fatigue, drunks or beggars. There are also individuals represented only from behind, as anonymous characters going on their way of existence, being unaware and indifferent to the by standing observer looking at the painting.

One of these very realistic characters, painted by Peter Brueghel the Elder, is of the action of yawning widely, in the work named De *Gaper.* It was taken by the English neurologist David Marsden as the paradigm of Brueghel’s Syndrome, in a medical article issued in 1976 about blepharospasm and oromandibular dystonia.

## Brueghel’s syndrome

David Marsden [1], in the title of his 1976 article on the subject, actually defined blepharospasm-oromandibular dystonia syndrome as ‘Brueghel’s syndrome’ and he did use the possessive eponym. But before him, in 1910, Henri Meige, a French neurologist, described approximately ten patients with involuntary closure of the eyelids. Blepharospasm was associated with involuntary contractions of the jaw muscles in only one of these patients. Over 60 years later, an American neurologist, George Paulson, from Columbus, Ohio reported three patients with blepharospasm and oromandibular dystonia and emphasized the probability of a common pathophysiological basis.

The terms ‘Meige’s syndrome’ and ‘Meige syndrome’ are often used by neurologists and other clinicians to describe the combination of blepharospasm and involuntary movements of the lower facial and/or masticatory muscles. This application of ‘Meige’s syndrome’ and other eponyms to the various forms of dystonia is problematic for a multitude of reasons. First of all, Meige, a physician, did not suffer from the syndrome that bears his name. Second, Meige was not the first person to describe the combination of blepharospasm and dystonia of other cranial muscles. Lastly, Meige’s or Meige syndrome could be confused with Meigs syndrome which is defined as the triad of a benign ovarian tumor, ascites and hydrothorax.

On the other hand, Brueghel was not a physician and his painting of a yawning subject has nothing to do with dystonia. Pieter Brueghel the Elder dropped the ‘h’ from his name in 1559, one year after painting the *De Gaper*. Brueghel the Younger was also a painter, further confounding a historically exact usage of this eponym. Dr. Gordon Gilbert neurologist in Saint Petersburg, Florida, suggested that the essential sign of ‘Brueghel syndrome’ is ‘a widely and dystonically opened jaw’ In reality, however, jaw-opening dystonia may occur in the setting of segmental, multifocal or generalized dystonia and may be associated with blepharospasm; thus application of ‘Breughel syndrome’ to these cases would be unnecessarily complicated and confusing.

In conclusion, the eponymic terms Meige syndrome and Brueghel syndrome are imprecise, have been used inconsistently for decades, and should be avoided in the classification of craniocervical dystonia. Mark Ledoux [2] underlines how the term segmental craniocervical dystonia faithfully incorporates various blepharospasm-plus sub phenotypes which appear to share common genetic and physiological underpinnings. Confident identification of genetic and environmental risk factors for segmental craniocervical dystonia may permit the developmental of better treatments which target pathways of cellular dysfunction within the central nervous system.


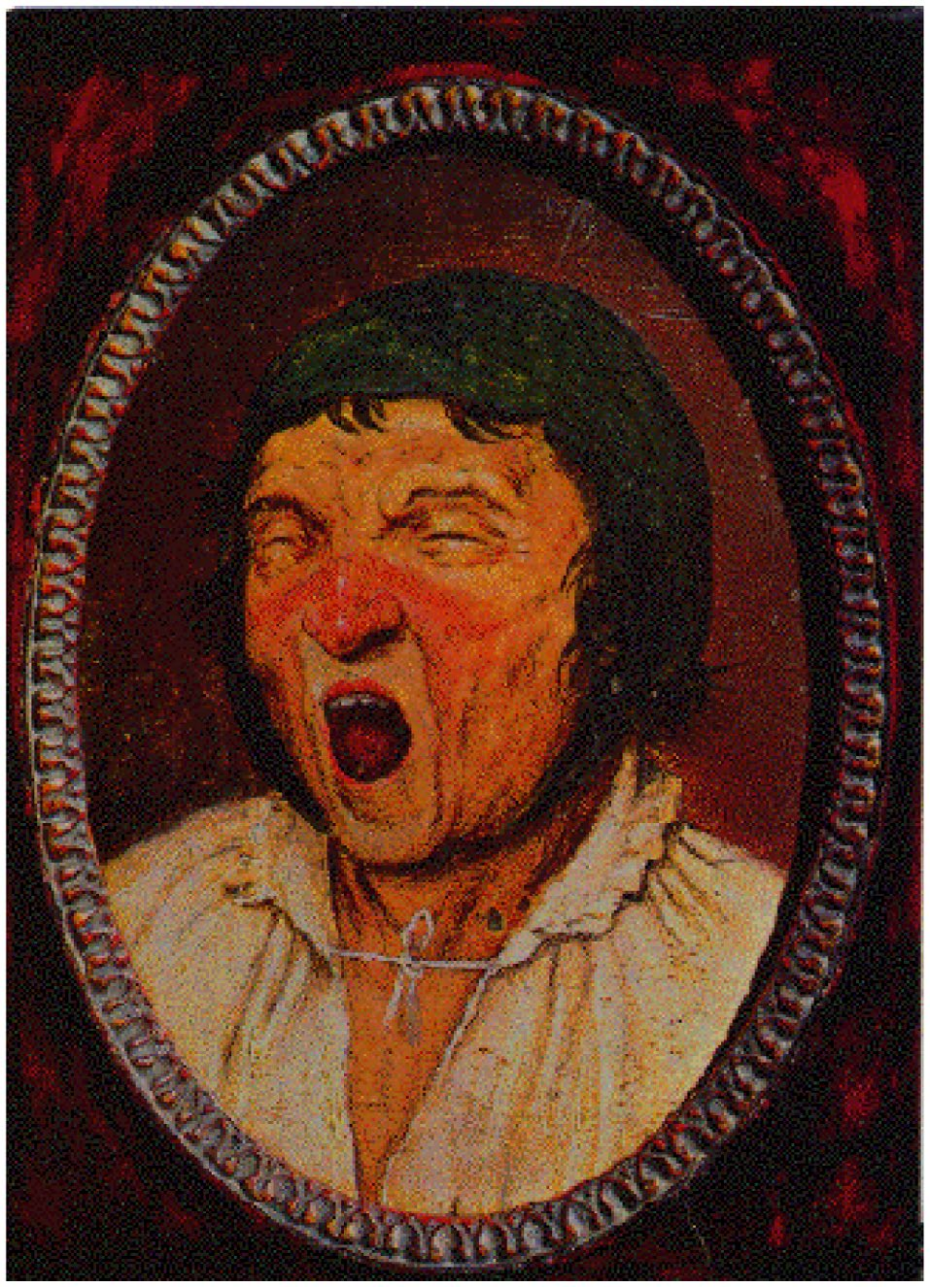
***Yawning man***

## Disclosure statement

No potential conflict of interest was reported by the author.
